# Subjective memory concerns and car collisions: A cross-sectional cohort study among older Japanese drivers

**DOI:** 10.1016/j.heliyon.2024.e33080

**Published:** 2024-06-19

**Authors:** Satoshi Kurita, Takehiko Doi, Kenji Harada, Masanori Morikawa, Chiharu Nishijima, Kazuya Fujii, Daisuke Kakita, Hiroyuki Shimada

**Affiliations:** a Department of Preventive Gerontology, Center for Gerontology and Social Science, Research Institute, National Center for Geriatrics and Gerontology, Obu, Japan; b School of Psychology, Faculty of Science, The University of New South Wales, Sydney, Australia; c Department of Medical Sciences, Medical Science Division, Graduate School of Medicine, Science and Technology, Shinshu University, Matsumoto, Japan

**Keywords:** Older driver, Cognitive function, Traffic incidents, Driving performance, Epidemiology

## Abstract

**Background:**

A previous study suggested older drivers with subjective memory concerns (SMC) had increased odds of experiencing car collisions, but whether SMC in different contexts and the number of SMC applicable items change this association is unknown. The aim of this study was to examine the association between SMC and car collisions among older drivers in Japan.

**Methods:**

This cross-sectional study was conducted using data from a Japanese community-based cohort study. Participants were community-dwelling older adults aged ≥60 years. SMC was assessed using five questions: 1) “Do you feel you have more problems with memory than most?” 2) “Do you have any difficulty with your memory?” 3) “Do you forget where you have left things more than you used to?” 4) “Do you forget the names of close friends or relatives?” and 5) “Do other people find you forgetful?” Participants were asked about their experiences with car collisions during the previous two years.

**Results:**

A total of 13,137 older drivers (72.1 ± 5.5 years old, and 43.6 % female) were analyzed. Cochran-Armitage trend test showed that as the number of SMC applicable items increased, the percentage of the experiences of car collisions significantly increased (6.8 %–15.8 %, P < 0.001). Logistic regression models showed each SMC question was associated with an increased odds ratio (OR) of car collisions (OR 1.26 to 1.71, all P < 0.001) after adjusting for confounding factors. As the number of SMC applicable items increased, the OR of car collisions significantly increased (OR 1.19 to 2.28, all P < 0.05, P for trend <0.001).

**Conclusions:**

This cross-sectional study among community-dwelling older drivers in Japan suggested each SMC question and the number of applicable items were associated with car collisions. SMC may be a sign of increased risk of traffic incidents for older drivers.

## Highlights

• This cross-sectional study included 13,137 drivers aged 60 years and over.
• The number of SMC items had significantly increased odds of car collisions.
• SMC may be a sign of increased risk of car collisions for older drivers.

## 1 Introduction

Car collisions by older drivers are likely to become severe as they age because of the decline of driving performance and decreased resistance against the impact of car collisions [1,2]. An analysis of road traffic crashes reports that victims with serious or fatal injuries caused by road traffic collisions were apt to increase as older drivers age [1,2]. Moreover, the number of older drivers is increasing as the population is aging. In Japan, there were 583 million older adults aged ≥75 years with a driver's license in 2019, and the number of older drivers is estimated to reach 760 million in 2024 [3]. This suggests that those who have a high risk of severe car collisions is increasing. There is a need to grasp the increased risk of car collisions at an early stage and prevent car collisions.

A recent cross-sectional observational study has found an association between subjective memory concerns (SMC) and traffic incidents among older drivers in Japan [4]. The association was independent from the factors of car accidents and dementia [5,6] including sleep problem, eye diseases, hearing problem, and objective cognitive impairment (OCI) assessed using cognitive tests of memory, attention, executive function, and processing speed. Numerous tests including Useful Field of View sub-test 2 and Multi-D [[7], [8], [9], [10], [11]] have been developed to substitute for the on-road driving test and identify impaired driving and have been compared [12,13]. However, SMC may be a useful measure because even older drivers can assess it by themselves. The above cross-sectional study has assessed SMC with five yes/no questions and finds that having at least one-applicable item was associated with the experience of a traffic incident [4]. Although the mechanism of this association and the influence of SMC on driving performance should be clarified, whether SMC in different contexts and the number of SMC applicable items changes this association with traffic incidents is unknown. Therefore, this study aimed to examine the association between SMC and traffic incidents among older drivers in Japan.

## 2 Material and methods

### 2.1 Participants

This cross-sectional study followed the Strengthening the Reporting of Observational Studies in Epidemiology (STROBE) reporting guidelines. This study was conducted using a dataset from a community-based cohort study, the National Center for Geriatrics and Gerontology-Study of Geriatric Syndromes (NCGG-SGS). The NCGG-SGS aims to establish a screening system for geriatric syndromes and validate evidence-based interventions for their prevention; this study has been described elsewhere [14]. During 2015–2018, 20,375 community-dwelling older adults aged ≥60 years participated in NCGG-SGS from Obu City, Takahama City, Tokai City, and Toyoake City in Aichi Prefecture, Japan. The surveys in Obu city and Takahama city were conducted in 2015–2016 and those in Toyoake city and Tokai city were conducted in 2017–2018. In the present study, participants were excluded if they were non-drivers (n = 5890); had a self-reported basic activity of daily living (BADL) disability (n = 11); a medical history that included stroke and dementia (n = 792); a general cognitive impairment (Mini-Mental State Examination score [MMSE] < 21; n = 130) [15]; or missing data for any of the variables used in our study (n = 415). A total of 13,137 participants were included in the analysis. All participants provided written informed consent. This study was conducted according to the Declaration of Helsinki guidelines. The National Center for Geriatrics and Gerontology research ethics committee approved the study protocol.

### 2.2 Measurement

#### 2.2.1 Subjective memory concerns

As with our previous study, SMC were assessed using five questions from a standardized memory loss question of the Geriatric Depression Scale (GDS) [16], the Cambridge Mental Disorders of the Elderly Examination (CAMDEX) questionnaire [17], and the Subjective Memory Complaints scale [18,19]: 1) “Do you feel you have more problems with memory than most?” 2) “Do you have any difficulty with your memory?” 3) “Do you forget where you have left things more than you used to?” 4) “Do you forget the names of close friends or relatives?” and 5) “Do other people find you forgetful?” Participants answered these questions “yes” or “no.”

#### 2.2.2 Car collision and near-miss traffic incidents

As with our previous study, the experience of car collisions and near-miss traffic incidents were set as the primary and secondary outcomes, respectively [4]. The experience of car collisions was assessed using a question: “Do you have a history of any car collision during the last 2 years?” Those who had a history of car collision(s) were asked details about the accident(s) by selecting the following answers: (1) accident occurred when you were driving and involved with persons on the road; (2) accident occurred when you were driving, and there was damage such as property damage; (3) accident involved another car, and you were culpable for less than half of the fault; (4) accident involved another car, and you were culpable for more than half of the fault; and (5) others. At least one “yes” answer was defined as the experience of car collisions.

The experiences of near-miss traffic incidents were assessed using questions concerning 12 situations while driving in the previous year. The participants were asked to answer “yes” or “no” to the 12 situations, such as almost hitting a pedestrian or other car (Table A1). At least one “yes” answer was defined as the experience of near-miss traffic incidents.

**Table 1:** Participants’ characteristics.

	Overall (n = 13,137)	No. of SMC applicable items						
		0 item (n = 4635)	1 item (n = 3487)	2 items (n = 2431)	3 items (n = 1555)	4 items (n = 807)	5 items (n = 222)	P
Age, mean (SD), year	72.1 (5.5)	71.7 (5.3)	72.0 (5.5)	72.2 (5.7)	72.5 (5.7)	72.7 (5.5)	73.0 (5.7)	<0.001^a^
Female, n (%)	5734 (43.6)	2045 (44.1)	1557 (44.7)	1072 (44.1)	686 (44.1)	313 (38.8)	61 (27.5)	<0.001^b^
Educational year, mean (SD)	12.0 (2.4)	12.1 (2.4)	12.1 (2.4)	12.0 (2.4)	11.9 (2.5)	11.8 (2.5)	12.2 (2.6)	0.003^a^
Eye disease, n (%)	4256 (32.4)	1368 (29.5)	1134 (32.5)	808 (33.2)	572 (36.8)	295 (36.6)	79 (35.6)	<0.001^b^
Hearing difficulty, %								
no	6639 (50.5)	2832 (61.1)	1798 (51.6)	1100 (45.2)	569 (36.6)	273 (33.8)	67 (30.2)	<0.001^c^
sometimes	4272 (32.5)	1319 (28.5)	1158 (33.2)	855 (35.2)	592 (38.1)	273 (33.8)	75 (33.8)	
yes	2226 (16.9)	484 (10.4)	531 (15.2)	476 (19.6)	394 (25.3)	261 (32.3)	80 (36.0)	
Medication use ≥5, %	2741 (20.9)	882 (19.0)	692 (19.8)	533 (21.9)	368 (23.7)	204 (25.3)	62 (27.9)	<0.001^b^
Sleep duration, %								
≥7 h	6207 (47.2)	2230 (48.1)	1712 (49.1)	1104 (45.4)	703 (45.2)	370 (45.8)	88 (39.6)	<0.001^c^
6.0–6.9 h	4302 (32.7)	1569 (33.9)	1109 (31.8)	816 (33.6)	483 (31.1)	251 (31.1)	74 (33.3)	
< 6 h	2628 (20.0)	836 (18.0)	666 (19.1)	511 (21.0)	369 (23.7)	186 (23.0)	60 (27.0)	
Excessive daytime sleepiness, %	1901 (14.5)	433 (9.3)	459 (13.2)	407 (16.7)	342 (22.0)	202 (25.0)	58 (26.1)	<0.001^b^
Driving time, mean (SD), min/day	51.0 (54.9)	53.5 (61.3)	49.3 (49.5)	51.5 (54.9)	48.4 (49.7)	49.3 (50.1)	46.8 (44.0)	0.002^a^
OCI, No. (%)	2941 (22.4)	951 (20.5)	748 (21.5)	539 (22.2)	399 (25.7)	235 (29.1)	69 (31.1)	<0.001^b^
Word memory^d^	1341 (10.2)	426 (9.2)	326 (9.3)	237 (9.7)	192 (12.3)	125 (15.5)	35 (15.8)	<0.001^b^
Trail making test – part A^d^	1051 (8.0)	346 (7.5)	281 (8.1)	180 (7.4)	143 (9.2)	79 (9.8)	22 (9.9)	0.008^b^
Trail making test – part B^d^	942 (7.2)	293 (6.3)	230 (6.6)	187 (7.7)	125 (8.0)	84 (10.4)	23 (10.4)	<0.001^b^
Symbol digit substitution test^d^	699 (5.3)	210 (4.5)	165 (4.7)	137 (5.6)	117 (7.5)	48 (5.9)	22 (9.9)	<0.001^b^

SMC, subjective memory concerns; OCI, objective cognitive impairment.

a Variables between groups were compared using one-way analysis of variance (ANOVA).

b Variables between groups were compared using Cochran-Armitage trend test.

c Variables between groups were compared using Pearson's χ^2^ test.

d Percentage of those who returned results lower than the standardized thresholds in each test.

#### 2.2.3 Confounding factors

Confounding factors were selected based on previous reviews concerning the factors of car accidents and dementia [5,6], which were the same as those selected in our previous study [4]. Sociodemographic data (age, sex, and education years), medical (eye disease, hearing difficulty, and the number of medications used), and lifestyle information (sleep duration, excessive daytime sleepiness [EDS], daily average driving time) were collected through face-to-face interviews. OCI was assessed in the four cognitive domains of memory, attention, executive function, and processing speed using the National Center for Geriatrics and Gerontology-Functional Assessment Tool (NCGG-FAT) [20]. Details about these variables are described in Table A2.

**Table 2:** Logistic regression model for the associations of each SMC item with car collisions and near-miss traffic incidents.

	Yes, n (% of overall participants)	Car collisions		Near-miss traffic incidents	
		OR (95%CI)	P	OR (95%CI)	P
Do you feel you have more problems with memory than most?	3937 (30.0)	1.26 (1.11**–**1.43)	<0.001	1.71 (1.57**–**1.85)	<0.001
Do you have any difficulty with your memory?	2287 (17.4)	1.34 (1.15**–**1.55)	<0.001	1.88 (1.71**–**2.08)	<0.001
Do you forget where you have left things more than you used to?	6219 (47.3)	1.71 (1.57**–**1.85)	<0.001	1.90 (1.76**–**2.04)	<0.001
Do you forget the names of close friends or relatives?	2917 (22.2)	1.34 (1.17**–**1.53)	<0.001	1.63 (1.49**–**1.78)	<0.001
Do other people find you forgetful?	1992 (15.2)	1.52 (1.30**–**1.76)	<0.001	1.84 (1.66**–**2.05)	<0.001

### 2.3 Statistical analysis

Comparison of participants' characteristics and outcomes by the number of SMC applicable items were conducted using the Cochran-Armitage trend test for binominal variables, Pearson's χ^2^-test for three categories variables, and one-way analysis of variance (ANOVA) for continuous variables. The associations of each SMC item and the number of SMC items with car collisions and near-miss traffic incidents were examined using binomial logistic regression models in a crude model and a fully adjusted model for all covariates. Odds ratios (OR) and 95 % confidence intervals (CI) referring to “no” answers for each SMC question and 0 items of SMC were calculated. As sub-analysis, to examine the interactions with confounding factors significantly associated with both the number of SMC applicable items and car collisions, we categorized the participants into eight groups by the number of SMC applicable items (0, 1, 2, ≥3 items) and the presence of the confounding factors. The associations among the eight groups and the outcomes were similarly examined using binomial logistic regression models in the fully adjusted model referred to individuals without SMC and confounding factors. The Cochran-Armitage trend test was conducted using R studio, and the other analyses were conducted using SPSS version 25 (IBM, New York City, NY, USA). The level of statistical significance was set at P < 0.05 for all analyses.

## 3 Results

**Fig. 1:**
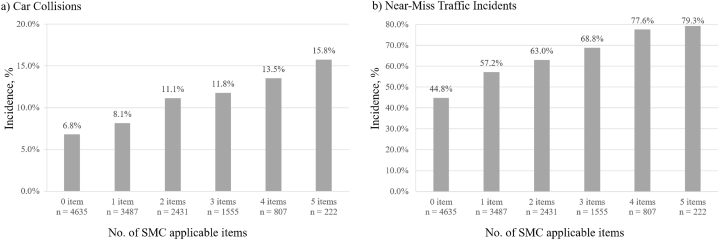
Comparison of (a) Car Collisions and (b) Near-Miss Traffic Incidents Among SMC Applicable Items Using the Cochran-Armitage Trend Test Both comparisons show P < 0.001. SMC, subjective memory concerns.

The 13,137 participants’ characteristics are summarized in Table 1 (72.1 ± 5.5 years old, 43.6 % female, and 12.0 ± 2.4 years of education). The number of participants with zero to five SMC applicable items were 4635 (35.3 %), 3487 (26.5 %), 2431 (18.5 %), 1555 (11.8 %), 807 (6.1 %), and 222 (1.7 %), respectively. The Cochran-Armitage trend test showed that as the number of SMC applicable items increased, the proportion of male, eye disease, medication use, EDS, and OCI increased (P < 0.001). The χ^2^ test for hearing difficulty and sleep duration showed significant differences among the number of SMC items (P < 0.001), and the proportion of “yes” answers for hearing difficulty and less than 6 h of sleep increased as the number of SMC applicable items increased. The Cochran-Armitage trend test showed the number of car collisions and near-miss traffic incidents increased as the number of SMC applicable items increased (P < 0.001, Fig. 1). This tendency was observed in each question concerning near-miss traffic incidents (Figure A1).

The results of the logistic regression model for the associations of each SMC question with car collisions and near-miss traffic incidents are summarized in Table 2. Each question was associated with reduced OR of car collisions and near-miss traffic incidents after the adjustment of covariates (car collisions: OR, 1.26 to 1.71; near-miss traffic incidents: OR, 1.63 to 1.90). In the logistic regression model of the association of the number of SMC items with car collisions and near-miss traffic incidents, an increased number of SMC items was associated with an increased OR after adjusting for covariates (car collisions: OR, 1.19 to 2.28, P for trend <0.001; near-miss traffic incidents: OR, 1.64 to 4.16, P for trend <0.001).

For the sub-analysis, we categorized the participants into eight groups by the number of SMC items and the presence of eye diseases, hearing difficulty, less than 6 h of sleep, EDS, and OCI, referring to the results from participants’ characteristics (Table 1) and the association between SMC applicable items and traffic incidents (Table 3). All analyses showed that SMC without the presence of these characteristics was significantly associated with an increased OR of traffic incidents, and SMC with the presence of these characteristics showed a relatively higher OR than that without (Figure A.2).

**Table 3:** Logistic regression model for the associations of the number of SMC applicable items with car collisions and near-miss traffic incidents.

	Car accidents				Near-miss traffic incidents			
	Crude		Adjusted		Crude		Adjusted	
	OR (95 % CI)	P	OR (95 % CI)	P	OR (95 % CI)	P	OR (95 % CI)	P
SMC applicable items (ref: 0 item)								
1 item	1.22 (1.03**–**1.44)	0.022	**1.19 (1.004ー1.41)**	**0.045**	1.64 (1.50**–**1.79)	<0.001	**1.64 (1.5ー1.79)**	**<0.001**
2 items	1.72 (1.45**–**2.04)	<0.001	**1.63 (1.37ー1.93)**	**<0.001**	2.10 (1.90**–**2.32)	<0.001	**2.04 (1.84ー2.26)**	**<0.001**
3 items	1.83 (1.51**–**2.22)	<0.001	**1.68 (1.38ー2.04)**	**<0.001**	2.71 (2.40**–**3.07)	<0.001	**2.62 (2.31ー2.97)**	**<0.001**
4 items	2.14 (1.70**–**2.70)	<0.001	**1.94 (1.53ー2.47)**	**<0.001**	4.26 (3.57**–**5.07)	<0.001	**4.01 (3.35ー4.79)**	**<0.001**
5 items	2.57 (1.76**–**3.75)	<0.001	**2.28 (1.55ー3.35)**	**<0.001**	4.71 (3.39**–**6.55)	<0.001	**4.16 (2.98ー5.81)**	**<0.001**
P for trend		<0.001		**<0.001**		<0.001		**<0.001**
Age			0.99 (0.98ー1.01)	0.268			**0.98 (0.98ー0.99)**	**<0.001**
Sex (ref: Female)			0.90 (0.79ー1.02)	0.097			**1.48 (1.38ー1.60)**	**<0.001**
Educational year			**1.04 (1.01ー1.07)**	**0.002**			**1.03 (1.01ー1.04)**	**0.001**
Eye diseases (ref: No)			**1.16 (1.02ー1.32)**	**0.027**			1.06 (0.98ー1.15)	0.144
Hearing difficulty (ref: No)								
Sometimes			**1.15 (1.01ー1.32)**	**0.040**			**1.22 (1.12ー1.32)**	**<0.001**
Yes			**1.26 (1.07ー1.49)**	**0.006**			**1.32 (1.19ー1.47)**	**<0.001**
Medication use (ref: <5)			1.09 (0.94ー1.26)	0.277			1.04 (0.95ー1.15)	0.358
Sleep duration (ref: ≥7 h)								
6.1–6.9 h			1.15 (0.995ー1.32)	0.058			**1.13 (1.04ー1.23)**	**0.003**
<6 h			**1.34 (1.14ー1.56)**	**<0.001**			1.05 (0.95ー1.16)	0.302
Excessive daytime sleepiness (ref: Less than a day in a week)			**1.27 (1.08ー1.48)**	**0.003**			**1.29 (1.16ー1.44)**	**<0.001**
Driving time per 30 min			**1.06 (1.04ー1.09)**	**<0.001**			**1.10 (1.08ー1.13)**	**<0.001**
OCI (ref: No)			1.12 (0.97ー1.29)	0.116			**0.88 (0.81ー0.96)**	**0.005**

SMC, subjective memory concerns; OCI, objective cognitive impairment.

## 4 Discussion

The present findings showed that among older Japanese drivers, each of the five SMC questions presenting different contexts had an association with the recent experience of car collisions and near-miss traffic incidents, and the OR of those outcomes increased as the number of SMC applicable items increased. Sub-analysis reinforced the robustness of the independent associations between SMC and those outcomes.

The research question has a potential theoretical paradox in that those with SMC could have forgotten their incidents. However, this possibility is fairly low and can be explained by the following points. First, those who had SMC in various contexts remembered those events; the present findings show that as the number of SMC applicable items increased so did the odds of experiencing an events. Second, the distribution of the incidence of outcomes, including each near-miss traffic incident by the number of SMC items, was similar (Fig. 1 and Figure A1). The higher the number of SMC items was, the higher the number of incidents was. Third, those with dementia and general cognitive impairments (MMSE score <21) were excluded from the study. Fourth, although not specifically analyzed in this study, it is well-known that car collisions are especially impactful events on drivers because of car damage requiring extensive repairs and traumatic memories requiring victim support. Therefore, it is unlikely that the study participants would forget their outcome events even when they had SMC and that similar distributions of outcomes by the number of SMC items could result despite the paradox.

Participants’ characteristics in this study showed that those with SMC in various contexts had a higher percentage of shorter sleep duration, EDS, eye disease, hearing difficulty, and OCI. These symptoms were reported as adverse factors for safe driving [5] and may partly explain the associations between SMC and traffic incidents. However, logistic models and sub-analysis suggested that the associations were independent from those adverse factors for safe driving. SMC may be independently associated with the increased risk of traffic incidents among older drivers; therefore, further research is needed to examine whether SMC can predict the occurrence of traffic incidents.

The different associations of SMC and OCI with traffic incidents, which was also observed in our previous study [4], are supported by a systematic review and meta-analysis that reported a small association between subjective and objective cognitive function [21,22]. In this study, SMC and OCI have different aspects and may affect driving performance differently. A systematic review evaluated the risk for driving safety depending on the severity of dementia syndromes and concluded that driving performance is severely impaired with moderate and severe dementia [23]. As for drivers with mild cognitive impairment (MCI) or mild dementia, this review concluded that they may still be able to drive under certain circumstances, depending on the characteristics of the underlying dementia, and recommend early checks for driving performance [23]. Because some studies could not detect the obvious differences in driving performance between drivers with MCI and normal cognition [[24], [25], [26], [27]], [[24], [25], [26], [27]] future studies should examine whether assessing the severity of SMC extends these findings.

Little research is concerned with the association between SMC and traffic incidents, but the present findings are supported by a cross-sectional study of driving behavior among 172 Japanese drivers aged 65 years or older [28]. The study assessed SMC using four questions, the same used in the present study excluding the GDS question, and compared self-reported driving behavior between older drivers with SMC (n = 137) and without SMC (n = 35). There were no significant differences of cognitive function between the two groups, but those with SMC were more apt to feel like another car will suddenly appear; overlook or do not notice in time pedestrians and obstacles; get lost or panic when driving on an unfamiliar road; and not be able to respond to dangerous situations immediately. These findings and the present findings suggest that older drivers with SMC are likely to have delayed reactions to other cars and pedestrians or have difficulty driving unfamiliar roads, which may increase the experiences of near-miss traffic incidents and car collisions. A growing number of studies on driving behavior propose research models [29], and cognition, prediction, judgment, and operation are considered as important. In terms of these driving tasks, SMC may be associated with traffic incidents by negatively influencing cognition, prediction, and judgement. Future research should examine the influence of SMC on these driving tasks.

Although understanding the mechanism of the direct association between SMC and traffic incidents seems difficult, this study might not have assessed certain intermediate factors. For example, the association between SMC and near-miss traffic incidents and delayed reactions to other cars and pedestrians [28] may reflect a decline in visuospatial ability. Visuospatial ability is a cognitive function that allows individuals to see objects clearly, reliably estimate their distance, and apply skills needed for rapid and effective decision-making while performing driving maneuvers [30]. Visuospatial ability supports human navigation in driving performance [30,31]. Because the participants’ characteristics (Table 1) suggested that the proportions of cognitive impairment in each domain increased according to the number of SMC applicable items, the proportion of visuospatial impairment may also similarly increase. The association between SMC and visuospatial ability should also be studied in the future.

SMC is assessed as a component of subjective cognitive decline (SCD); therefore, examining the association between SCD and traffic incidents may extend the present findings. SCD is defined as the self-experienced, persistent decline in cognitive capacity compared with a previous normal cognitive status and is unrelated to an acute event in the context of normal performance on objective cognitive measures [32]. Although a gold standard assessment tool for SCD has not been established [32], SCD concerns not only memory but also other cognitive domains, including attention and executive function [33].

The strength of this study is that it is the first to examine a detailed association between SMC and traffic incidents. These findings could extend previous findings concerning the association of motoric cognitive risk syndrome with traffic incidents [4]. Nevertheless, this study has some limitations. First, the self-report data collection method may have resulted in recall bias. Some studies have reported that near-miss traffic incidents are easily forgotten [34]. Second, this study has a cross-sectional study design and cannot mention the causal relationships of SMC with traffic incidents. Additionally, this study design could not confirm whether the participant had SMC at the time of the car accidents or near-miss traffic incidents. Third, the excluded missing data (n = 415) or residual confounders after grouping by the number of SMC applicable items alongside the presence of confounding factors may have influenced the results.

## 5 Conclusion

In this cross-sectional study of community-dwelling older Japanese drivers, five SMC questions presenting different contexts were respectively associated with car collisions and near-miss traffic incidents. As the number of SMC applicable items increased, the OR of those outcomes increased. Further studies examining different settings or using a longitudinal design are needed to clarify whether SMC has the predictivity of future traffic incidents. In addition, future studies should examine the association of SMC with driving performance to clarify the mechanism of the present findings.

## Funding sources

This work was supported by the 10.13039/100009619Japan Agency for Medical Research and Development (grant no. 15dk0207019h0001, 15dk0107003h0003, 15dk0207004h0203, 18le0110004h0002, and 18dk0110021h0003); Research Funding for Longevity Sciences from the 10.13039/501100007312National Center for Geriatrics and Gerontology (grant no. 26-33, 27-22, 28–30, 29–31, and 29–42); Health and Labour Sciences research grants from the Japanese 10.13039/100009647Ministry of Health, Labour, and Welfare (grant no. H29-ninchisho-ippan-002), the 10.13039/501100001691Japan Society for the Promotion of Science KAKENHI (grant no. 18H03185 and 22K11846), and funds of the Obu City Local Government.

## Data statements

The datasets used and/or analyzed during the present study are available from the corresponding author on reasonable request.

## CRediT authorship contribution statement

**Satoshi Kurita:** Conceptualization, Formal analysis, Funding acquisition, Investigation, Writing – original draft. **Takehiko Doi:** Formal analysis, Funding acquisition, Investigation, Supervision, Writing – review & editing. **Kenji Harada:** Writing – review & editing. **Masanori Morikawa:** Writing – review & editing. **Chiharu Nishijima:** Writing – review & editing. **Kazuya Fujii:** Writing – review & editing. **Daisuke Kakita:** Writing – review & editing. **Hiroyuki Shimada:** Funding acquisition, Project administration, Supervision, Writing – review & editing.

## Declaration of competing interest

None.
