# Supplementary material for: Subjective memory concerns and car collisions: A cross-sectional cohort study among older Japanese drivers
Source: Heliyon. 2024 Jun 19;10(12):e33080. doi: 10.1016/j.heliyon.2024.e33080 (PMC11253256; doi:10.1016/j.heliyon.2024.e33080)
Supplement: Multimedia component 1 [file mmc1.docx]

**Table A.1. The 12 Items of Questions of Near-Miss Traffic Incident**

1. When going from a stop line, I almost hit someone coming from a different direction.

2. When attempting to turn right, I almost hit a car coming straight on.

3. When attempting to turn right, I almost hit a pedestrian or bicycle.

4. When attempting to turn left, I almost hit a pedestrian or bicycle.

5. I drifted greatly into the oncoming lane and almost collided head-on with another vehicle.

6. When changing lanes, I almost collided with another vehicle.

7. I almost collided with a vehicle in front of me.

8. I almost struck a bicycle, motorcycle, other vehicle I was overtaking or passing.

9. I made a mistake while stepping on the accelerator or brake.

10. When starting on a hill, I almost hit another vehicle or obstacle (including living things).

11. When backing up to park, I almost hit another vehicle or obstacle (including living things).

12. When I entered a store parking lot from the road, I almost ran up on the curb of the sidewalk.

**Table A.2. Confounding Factors**

| **Item** | **Assessing** |
| --- | --- |
| Eye diseases | The presence of glaucoma, cataract, or other diseases. |
| Hearing difficulty | A question from the Hearing Handicap Inventory for the Elderly Screening Version (HHIE-S): “Do you have difficulty hearing when someone speaks in a whisper?” The responses were yes, sometimes, or no.^1^ |
| Polypharmacy | Five or more medications used was identified as Polypharmacy.^2^ |
| Sleep duration | Calculation by the difference between self-reported usual sleep and wake times. The time was categorized into three levels (≥7 hours, 6.0–6.9 hours, or <6 hours) referring to a literature on acute sleep deprivation and culpable motor vehicle collision involvement.^3^ |
| Excessive daytime sleepiness | The question “How often do you experience daytime sleepiness which causes difficulty staying awake?” with the following options in response: “3 or more days a week,” “1 or 2 days a week,” “less than a day a week,” and “never.” These responses were divided into “1 or more days in a week” and “less than a day a week.” |
| Average daily driving time for a week | Calculated from (self-reported driving days in a week × driving time in a day)/ 7. |
| Objective cognitive impairment | Neuropsychological tests using the National Center for Geriatrics and Gerontology-Functional Assessment Tool (NCGG-FAT), which has been described elsewhere.^4^ Four cognitive domain tests were conducted: memory (word list memory-I [immediate recognition] and word list memory-II [delayed recall]), attention (an electronic-tablet version of the Trail Making Test [TMT] part A), executive function (an electronic-tablet version of the TMT-part B), and processing speed (an electronic-tablet version of the Symbol Digit Substitution Test). Those who returned results lower than the standardized thresholds for one or more tests in the NCGG-FAT were defined as having objective cognitive impairment. |

**References**

1. Ventry IM, Weinstein BE. The hearing handicap inventory for the elderly: a new tool. *Ear Hear.* 1982;3(3):128-134.

2. Gnjidic D, Hilmer SN, Blyth FM, et al. Polypharmacy cutoff and outcomes: five or more medicines were used to identify community-dwelling older men at risk of different adverse outcomes. *J Clin Epidemiol.* 2012;65(9):989-995.

3. Tefft BC. Acute sleep deprivation and culpable motor vehicle crash involvement. *Sleep.* 2018;41(10).

4. Makizako H, Shimada H, Park H, et al. Evaluation of multidimensional neurocognitive function using a tablet personal computer: test-retest reliability and validity in community-dwelling older adults. *Geriatr Gerontol Int.* 2013;13(4):860-866.
