# Supplementary material for: Subjective memory concerns and car collisions: A cross-sectional cohort study among older Japanese drivers
Source: Heliyon. 2024 Jun 19;10(12):e33080. doi: 10.1016/j.heliyon.2024.e33080 (PMC11253256; doi:10.1016/j.heliyon.2024.e33080)
Supplement: Multimedia component 2 [file mmc2.pptx]

## Slide 1
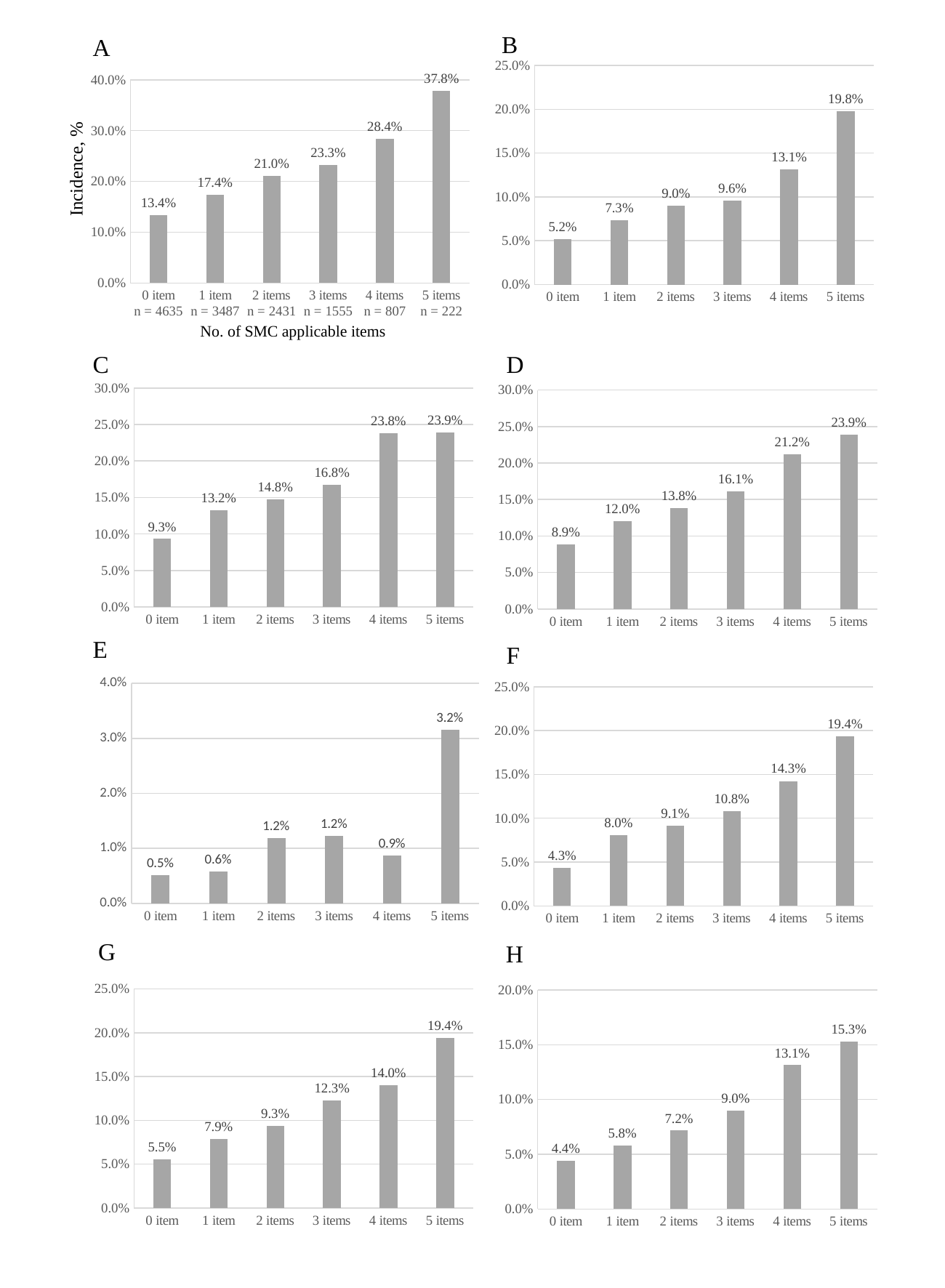

B
A
### Chart
| Category | ヒヤリハット経験あり |
|---|---|
| 0 item | 0.05156418554476807 |
| 1 item | 0.07341554344708918 |
| 2 items | 0.08967503085150144 |
| 3 items | 0.09581993569131834 |
| 4 items | 0.13135068153655513 |
| 5 items | 0.1981981981981982 |
### Chart
| Category | ヒヤリハット経験あり |
|---|---|
| 0 item
n = 4635 | 0.133764832793959 |
| 1 item
n = 3487 | 0.17350157728706622 |
| 2 items
n = 2431 | 0.2102015631427396 |
| 3 items
n = 1555 | 0.23279742765273312 |
| 4 items
n = 807 | 0.28376703841387857 |
| 5 items
n = 222 | 0.3783783783783784 |Incidence, %
No. of SMC applicable items
C
D
### Chart
| Category | ヒヤリハット経験あり |
|---|---|
| 0 item | 0.09320388349514562 |
| 1 item | 0.13220533409807858 |
| 2 items | 0.1476758535582065 |
| 3 items | 0.1678456591639871 |
| 4 items | 0.2379182156133829 |
| 5 items | 0.23873873873873877 |
### Chart
| Category | ヒヤリハット経験あり |
|---|---|
| 0 item | 0.08888888888888889 |
| 1 item | 0.11987381703470032 |
| 2 items | 0.13780337309749074 |
| 3 items | 0.1607717041800643 |
| 4 items | 0.21189591078066916 |
| 5 items | 0.23873873873873877 |E
F
### Chart
| Category | ヒヤリハット経験あり |
|---|---|
| 0 item | 0.00517799352750809 |
| 1 item | 0.005735589331803843 |
| 2 items | 0.011929247223364869 |
| 3 items | 0.012218649517684888 |
| 4 items | 0.008674101610904586 |
| 5 items | 0.03153153153153153 |
### Chart
| Category | ヒヤリハット経験あり |
|---|---|
| 0 item | 0.04314994606256742 |
| 1 item | 0.0802982506452538 |
| 2 items | 0.09132044426162073 |
| 3 items | 0.1080385852090032 |
| 4 items | 0.14250309789343246 |
| 5 items | 0.19369369369369369 |G
H
### Chart
| Category | ヒヤリハット経験あり |
|---|---|
| 0 item | 0.05501618122977346 |
| 1 item | 0.07886435331230283 |
| 2 items | 0.09337721102426984 |
| 3 items | 0.12282958199356914 |
| 4 items | 0.14002478314745972 |
| 5 items | 0.19369369369369369 |
### Chart
| Category | ヒヤリハット経験あり |
|---|---|
| 0 item | 0.04422869471413161 |
| 1 item | 0.05821623171780901 |
| 2 items | 0.07157548334018922 |
| 3 items | 0.09003215434083602 |
| 4 items | 0.13135068153655513 |
| 5 items | 0.15315315315315314 |

## Slide 2
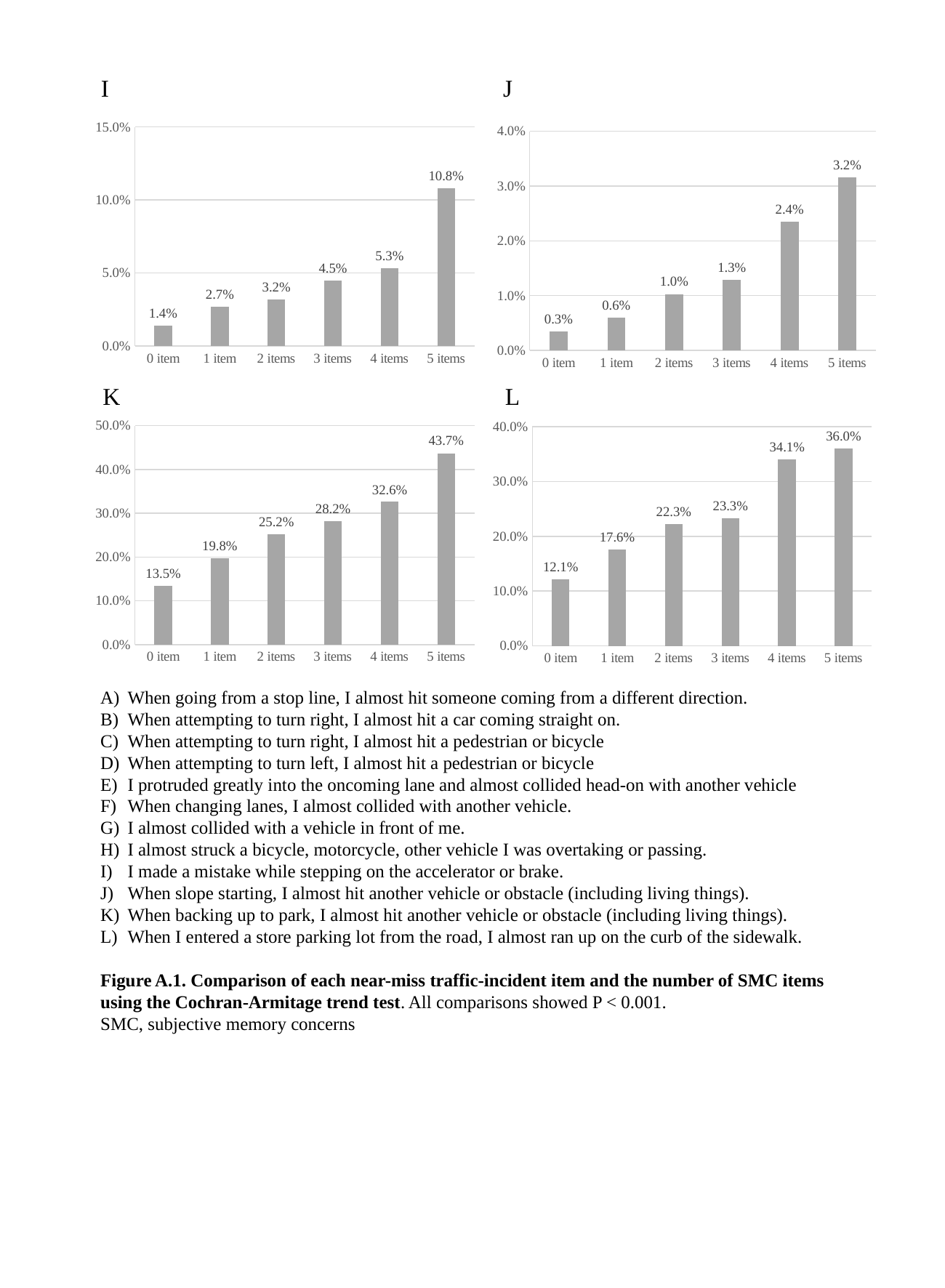

I
J
### Chart
| Category | ヒヤリハット経験あり |
|---|---|
| 0 item | 0.013807982740021575 |
| 1 item | 0.02667049039288787 |
| 2 items | 0.03167420814479638 |
| 3 items | 0.04501607717041801 |
| 4 items | 0.053283767038413886 |
| 5 items | 0.1081081081081081 |
### Chart
| Category | ヒヤリハット経験あり |
|---|---|
| 0 item | 0.003451995685005394 |
| 1 item | 0.006022368798394035 |
| 2 items | 0.010283833813245578 |
| 3 items | 0.012861736334405145 |
| 4 items | 0.023543990086741014 |
| 5 items | 0.03153153153153153 |K
L
### Chart
| Category | ヒヤリハット経験あり |
|---|---|
| 0 item | 0.1348435814455232 |
| 1 item | 0.19787783194723257 |
| 2 items | 0.25215960510078156 |
| 3 items | 0.28167202572347266 |
| 4 items | 0.32589838909541513 |
| 5 items | 0.4369369369369369 |
### Chart
| Category | ヒヤリハット経験あり |
|---|---|
| 0 item | 0.12125134843581446 |
| 1 item | 0.17608259248637798 |
| 2 items | 0.22254216371863433 |
| 3 items | 0.2334405144694534 |
| 4 items | 0.3407682775712516 |
| 5 items | 0.3603603603603604 |When going from a stop line, I almost hit someone coming from a different direction.
When attempting to turn right, I almost hit a car coming straight on.
When attempting to turn right, I almost hit a pedestrian or bicycle
When attempting to turn left, I almost hit a pedestrian or bicycle
I protruded greatly into the oncoming lane and almost collided head-on with another vehicle
When changing lanes, I almost collided with another vehicle.
I almost collided with a vehicle in front of me.
I almost struck a bicycle, motorcycle, other vehicle I was overtaking or passing.
I made a mistake while stepping on the accelerator or brake.
When slope starting, I almost hit another vehicle or obstacle (including living things).
When backing up to park, I almost hit another vehicle or obstacle (including living things).
When I entered a store parking lot from the road, I almost ran up on the curb of the sidewalk.
Figure A.1. Comparison of each near-miss traffic-incident item and the number of SMC items using the Cochran-Armitage trend test. All comparisons showed P < 0.001.
SMC, subjective memory concerns

## Slide 3
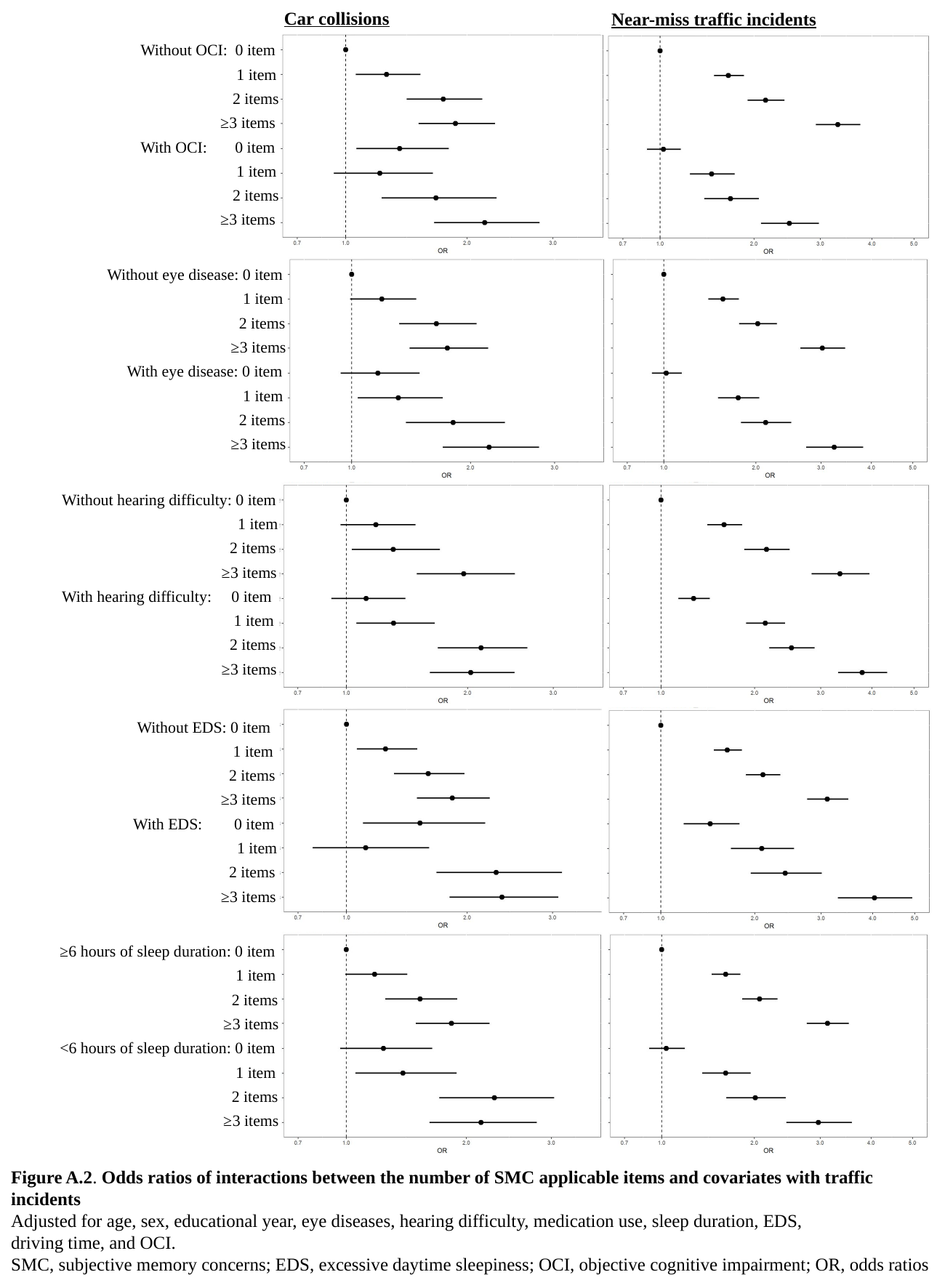

Car collisions
Near-miss traffic incidents
Without OCI: 0 item
 1 item
 2 items
 ≥3 items
With OCI: 0 item
 1 item
 2 items
 ≥3 items
 Without eye disease: 0 item
 1 item
 2 items
 ≥3 items
 With eye disease: 0 item
 1 item
 2 items
 ≥3 items
 Without hearing difficulty: 0 item
 1 item
 2 items
 ≥3 items
 With hearing difficulty: 0 item
 1 item
 2 items
 ≥3 items
 Without EDS: 0 item
 1 item
 2 items
 ≥3 items
With EDS: 0 item
 1 item
 2 items
 ≥3 items
 ≥6 hours of sleep duration: 0 item
 1 item
 2 items
 ≥3 items
 <6 hours of sleep duration: 0 item
 1 item
 2 items
 ≥3 items
Figure A.2. Odds ratios of interactions between the number of SMC applicable items and covariates with traffic incidents
Adjusted for age, sex, educational year, eye diseases, hearing difficulty, medication use, sleep duration, EDS,
driving time, and OCI.
SMC, subjective memory concerns; EDS, excessive daytime sleepiness; OCI, objective cognitive impairment; OR, odds ratios
